# HEP^®^ (Homeostasis-Enrichment-Plasticity) Approach Changes Sensory–Motor Development Trajectory and Improves Parental Goals: A Single Subject Study of an Infant with Hemiparetic Cerebral Palsy and Twin Anemia Polycythemia Sequence (TAPS)

**DOI:** 10.3390/children11070876

**Published:** 2024-07-19

**Authors:** Aymen Balikci, Teresa A. May-Benson, Gamze Cagla Sirma, Gul Ilbay

**Affiliations:** 1 Sense On, Ltd., Istanbul 34810, Türkiye; 2 TMB Educational Enterprises, LLC., Norristown, PA 19401, USA; tmb@tmbeducation.com; 3 Department of Occupational Therapy, Faculty of Health Sciences, Fenerbahçe University, Istanbul 34758, Türkiye; gamze.dirgen@fbu.edu.tr; 4 Department of Physiology, Faculty of Medicine, Kocaeli University, Kocaeli 41001, Türkiye; gilbay@kocaeli.edu.tr

**Keywords:** cerebral palsy, twin anemia polycythemia sequence, early intervention, enriched environment

## Abstract

Background: Early intervention (EI) for infants identified as being at high risk for cerebral palsy (CP), or who have been diagnosed with it, is critical for promotion of postnatal brain organization. The aim of this study was to explore the effectiveness of the Homeostasis-Enrichment-Plasticity (HEP) Approach, which is a contemporary EI model that applies the key principles of enriched environment paradigms and neuronal plasticity from experimental animal studies to ecological theories of human development on the motor development, sensory functions, and parental goals of an infant with twin anemia polycythemia sequence (TAPS) and CP. Methods: An AB phase with follow-up single case study design which consisted of multiple baseline assessments with the Peabody Developmental Motor Scales-2 (PDMS-2) and the Test of Sensory Functions in Infants (TSFI) was used. Non-overlapping confidence intervals analysis was used for pre–post PDMS-2 scores. The measurement of progress toward goals and objectives was conducted using the Goal Attainment Scale (GAS). The HEP Approach intervention consisted of 12 one-hour sessions implemented over a period of 3 months, where a physical therapist provided weekly clinic-based parental coaching. Results: Results found a stable baseline during Phase A and improvement in response to the HEP Approach intervention during Phase B in both the PDMS-2 and TSFI according to 2SD Band analysis. The confidence intervals for the PDMS-2 scores also indicated a significant improvement after HEP intervention. The scores for both the PDMS-2 and the TSFI were consistent or showed improvement throughout the Follow-Up phase. A GAS t-score of 77.14 indicated that the infant exceeded intervention goal expectations. Conclusions: Although our findings suggest that the HEP Approach intervention has promise in enhancing sensory functions, motor skill outcomes, and parental goals in an infant with TAPS and CP, further research is required to validate and apply these results more broadly.

## 1. Introduction

Cerebral palsy (CP) is a term for a group of non-progressive neurodevelopmental disorders resulting from interference in brain development [1,2]. CP is a movement and postural disorder that results in activity limitations [3]. However, in recent years, clinicians have also appreciated that CP may also affect sensation, perception, cognition, communication, and behavior [4,5,6]. A recent global prevalence study found that CP prevalence in high-income countries is 1.6 per 1000 live births and is significantly higher in low- and middle-income countries [2].

The etiology of CP varies, and risk factors include placental abruption, uterine rupture, cord prolapse, intrauterine exposure to infections, prematurity, congenital anomalies, maternal fever during delivery, hypoxic ischemia, cerebrovascular insults during pregnancy, intrauterine growth retardation, complications of multiple gestations; and in infancy, accidental and non-accidental brain injury. Emerging evidence also suggests a possible interaction between genetic vulnerabilities and environmental pollutants in understanding the etiology of CP [1,2].

Additionally, studies conducted in recent years reveal that twin anemia polycythemia sequence (TAPS) can occur in monochorionic twins who share a single placenta and is caused by unbalanced inter-twin blood transfusion as a result of blood flowing bidirectionally between the two fetuses with inter-twin anastomoses [7,8]. TAPS is characterized by a high rate of neurodevelopmental impairment such as CP [8,9]. Moreover, it is suggested that the most pervasive impact of TAPS occurs in childhood, Therefore, early screening and regular long-term follow-up are essential for the care of TAPS twins [9].

Early intervention (EI) for infants at high risk for CP or diagnosed with CP is essential to promote postnatal brain organization [10,11]. EI is defined as a system of services provided to children from birth until 3 years of age who are identified with a specific developmental disorder or at risk for delays in one or more aspects of functioning [12]. EI services have been available in countries worldwide for decades, with the assumption that they lead to positive long-term outcomes for the child and improve the well-being of the family. However, the impact of EI on developmental outcomes is not consistently reported in the literature [12,13]. The types of interventions provided, insufficient intervention doses, and concern that the intervention was not delivered early enough have been posited as possible reasons for the inconsistent effectiveness of early intervention on developmental outcomes [12,13,14]. 

Before the last decade, EI for infants with CP focused on improving body structures and functions, such as muscle tone or movement patterns, with an expectation that these changes will improve children’s function in daily tasks. However, limited evidence supports their efficacy [4,11,15]. Current EI trends, which now appreciate the impact of personal and environmental factors on the development and health of the child, support a change in focus from this traditional impairment-based intervention approach. EI is now aligned with the biopsychosocial model supported by the International Classification of Functioning, Disability and Health [16], which is focused on enhancing children’s participation in preferred activities, regardless of emerging or persistent deficits. Moreover, it is suggested that personal factors within the surrounding environmental context that influence developmental outcomes should be a key target of EI [11,17,18]. In summary, recent research suggests that EI should focus on targeted play activities, family involvement, and environmental enrichment [11,16,19,20]. Enriched environment (EE) is an experimental paradigm that significantly enhances recovery after brain injury by experience-dependent neuroplasticity, the primary mechanism used by rehabilitation professionals to promote improved function [4,19,21,22,23]. Interest in EE-based EI models for infants at risk for developmental delays, including CP, has increased over the past decade [4,19]. Moreover, an increasing number of studies suggest that EE-based EI is an evidence-based model that improves the functional capacity of children at risk of or diagnosed with CP [19,24,25,26]. 

Experimental studies have also demonstrated that EE can mitigate brain damage and improve motor outcomes in rat models of CP [27,28,29]. A study by Marques et al. (2014) revealed that stimulus increments provided by EE may successfully prevent the development of motor impairments and histological abnormalities in a rat model CP [27]. Additionally, another study showed that EE effectively protects the motor impairments observed in a rat model of CP, as measured on postnatal day 31 [28]. A recent comprehensive review and meta-analysis demonstrated that EE significantly improved the behavioral and histological abnormalities resulting from the lesion [29]. These findings indicate that EE may be an effective approach for managing newborn hypoxia-ischemia [28], a recognized underlying factor for cerebral palsy [30]. 

Multiple studies have investigated the mechanism by which EE affects neonatal hypoxic-ischemic conditions and behavioral outcomes. A study conducted by Xie et al. (2020) proposed that exposure to an EE facilitated the recovery of blood flow in the damaged area of the brain and improved the coordination between functional activation and changes in blood flow [31]. These mechanisms may explain the neuroprotective effects of EE following an ischemic event. Another study stated that the neuroprotective effects of EE might be accomplished by promoting the transformation of M2 microglia through activation of the PI3K/AKT/GSK3β signaling pathway. This leads to the inhibition of inflammation, creating a favorable environment for the maturation of oligodendrocytes and the process of remyelination [32]. Notably, research has demonstrated that EE can reduce the amounts of pro-inflammatory chemicals, such as tumor necrosis factor-alpha and interleukin 1b, in the cortex and hippocampus following a brain injury. Considering the increased expression of these variables caused during brain injury, the effect of EE may reduce the extent of damage associated with secondary injury [33,34]. Researchers also proposed that in chronic hypoxic-ischemic brain injury, EE upregulates fibroblast growth factor-2 to promote endogenous angiogenesis and neurobehavioral functioning [35]. In their study, Song et al. (2021) reported that EE can increase the expression of the Cav 2.1 channel and presynaptic proteins associated with the synaptic vesicle cycle and neurotransmitter release [36]. They suggested that these changes may contribute to the improvement of motor and cognitive functions in individuals with hypoxic-ischemic encephalopathy. Research has shown that EE may lead to structural changes by increasing the release of trophic factors such as vascular endothelial growth factor and brain-derived neurotrophic factor under brain-injured conditions [37,38]. These factors promote cell survival and adaptation, while also stimulating the upregulation of transcription factors that regulate the synthesis of proteins involved in plasticity [34]. Exposure to EE can lead to an elevation in levels of neurotransmitters like noradrenaline and dopamine, as well as an increase in the expression of NMDA receptors and brain metabolic activity. These factors, which are affected by brain injury and are associated with motor and cognitive dysfunction, could potentially be regulated by exposure to EE [34,39]. Considering the limited understanding of the impacts of EE, it is evident that EE promotes new neuron growth, development of dendritic branches, increased density of spines, and the expression of growth factors. 

Current EE-based EI models fail to comprehensively address multiple areas of development in children in EI, specifically areas such as regulation/homeostasis, active self-directed exploration, and continuous engagement with the environment. Further, these programs do not treat the child as a holistic being. The Homeostasis-Enrichment-Plasticity (HEP) Approach, a recent EI model, was developed to address these limitations and translate the principles of EE and ecological theories of development to clinical physical and occupational therapy practice. Unlike current models, the HEP Approach comprehensively applies the core principles of EE paradigms and neural plasticity used in experimental animal studies to the context of ecological theories of human development (e.g., sensory integration, dynamic systems, and perception–action), and emphasizes the fundamental importance of physiological homeostasis in the child as well as the child’s relationship with the parents [4]. Areas of function targeted by this novel approach include sensory functioning and self-regulation, motor skills performance and planning, and functional performance.

This study aims to investigate the effectiveness of the HEP intervention approach on the sensory functions, motor skills, and parental goals of an infant with TAPS and CP.

## 2. Methods

### 2.1. Participant

Z is a 9-month-old female who was born at term as an identical twin with a birth weight of 2775 g. She was diagnosed with twin anemia polycythemia sequence (TAPS) and required a 30-day stay in the Neonatal Intensive Care Unit (NICU) for TAPS-specific medical interventions. After the NICU period, baby Z was discharged home. When the twins were about 6 months old, their parents noticed differences between Z and her twin in tummy time, sitting, and right-hand use. The pediatrician referred the family to a pediatric neurologist for a screening evaluation. When Z was 9 months old, she was diagnosed with hemiparetic cerebral palsy according to a comprehensive neurological evaluation and MRI results. After diagnosis, the family was referred to the local hospital’s physical therapy and rehabilitation service for physiotherapy. A traditional physical therapy intervention program was followed, including passive exercises, weight shifting, positioning, and trunk control activities, for one session per week. During the same period, the family was referred to the first author for a second opinion evaluation. A comprehensive assessment by the first author found that the infant had good self-regulation and was ready for social engagement. However, the infant could not sit independently, stand on her feet, or do a four-point crawl. Her right hand was always fisted which did not enable functional hand usage. Furthermore, an ecological perspective observation [40] showed that the infant did not explore support surfaces with her hands and feet in a seated position, and limited exploration of support surfaces occurred in prone and supine positions (less exploration with right extremities was observed). Also, the infant was limited in exploration of her body and objects using her hands in all positions. As a result of this evaluation, it was suggested to the parents that the most appropriate intervention would be an ecologically based enriched environment approach (the HEP Approach intervention program). As a first option, parents chose to follow the traditional intervention program at the hospital which was closer to their home. However, the family agreed to monthly evaluations from the first author, and to participate in the suggested intervention after three months as a part of this case study.

### 2.2. Study Design

An AB phase change without reversal single case study design was used to evaluate the potential effectiveness of the HEP Approach on measures of sensory functions, motor development, and parental goals [41,42]. 

A reversal design was not used as the functions under examination were not expected to regress to baseline with the withdrawal of the intervention. Four comprehensive assessments were completed at Pre-Phase A, Pre-Phase B, Post-Phase B, and at Follow-Up. Selected tests were completed every two weeks or monthly during baseline and intervention.

Phase A: A comprehensive assessment was completed using observation, parent interview, the Test of Sensory Functions in Infants (TSFI), and the Peabody Developmental Motor Scales-2 (PDMS-2). Between the ages of 9 and 12 months, Z participated in a traditional physiotherapy intervention program provided independently of the study authors at the local hospital. The program included massage, passive range of motion, rolling and crawling exercises, prone positioning, weight-bearing, and trunk control activities on the ball. Monthly evaluation of sensory and motor development was provided using the outcome measures of PDMS-2 and the TSFI.

Phase B: The comprehensive assessment completed at 9 months using observation, parent interview, TSFI, and the PDMS-2 was repeated when Z was 12 months of age after the conclusion of traditional physical therapy and before initiation of the HEP Approach intervention. Functional goals for the HEP Approach intervention using Goal Attainment Scales (GAS) were set together with the parents. Z then engaged in the HEP Approach intervention for 3 months between 12 and 15 months of age. Motor development was assessed twice monthly with the PDMS-2 and sensory functioning once monthly with the TSFI. Comprehensive assessment was re-administered at the end of the intervention. Z’s goal attainment scales, created before the intervention, were rated post-intervention by her parents. 

Follow-Up: One Follow-Up comprehensive assessment (the same as Phase A and B) was completed after the HEP Approach intervention, at 19 months of age. In addition, shared videos via WhatsApp showed that the mother was trying new activities that enabled the infant to explore objects, support surfaces, and movement possibilities.

### 2.3. Outcome Measures

Peabody Developmental Motor Scales—2 (PDMS-2). PDMS-2 was selected as a measure to evaluate motor development as it is consistently used in early intervention research as a primary outcome measure for motor performance. It consists of six subtests that evaluate the gross and fine motor abilities of children from infancy to 5 years of age. Each of the PDMS-2 subtests contributes to the calculation of a Total Motor Quotient (TMQ). Typically, this score is regarded as the most accurate assessment of general motor skills. Furthermore, every subtest makes a distinct contribution to either the Gross Motor Quotient (GMQ) or the Fine Motor Quotient (FMQ) score. The GMQ assesses the capacity to utilize the major muscular systems for locomotion, maintain a steady posture while stationary, respond instinctively to changes in the environment, and successfully grasp and release things. The FMQ assesses a child’s manual dexterity in and capacity to handle objects, grasp items, stack blocks, draw shapes, and complete object manipulation tasks using hands and arms. Testing requires approximately 45 to 60 min, although it may be shorter for infants [43,44]. The PDMS-2 is recognized as a reliable and valid tool for evaluating the motor development of premature infants [44,45].

Test of Sensory Functions in Infants (TSFI). The TSFI was selected as an age-appropriate measure of sensory processing as it is the only standardized direct assessment measure for this function available for infants. It is a therapist-administered standard performance test of sensory functioning for infants between the ages of 4 and 18 months. The TSFI is recommended for infants with or at risk for regulatory disorders, developmental delays, and sensory processing disorder. The test consists of 24 items divided into five sub-tests of sensory processing and reactivity. These sub-tests are Reactivity to Deep Touch Pressure, Adaptive Motor Functions, Visual–Tactile Integration, Ocular–Motor Control, and Reactivity to Vestibular Stimulation. In the normal distribution curve, scores above −1 SD are scored as “Normal”, scores between −1 SD and −2 SD are scored as “At risk”, and scores below −2 SD are scored as “Deficient” [46,47]. 

Goal Attainment Scaling. (GAS). Goal Attainment Scales were used to report progress on individualized functional goals. GAS is the preferred goal-setting methodology for documenting individualized change during and after intervention in clinical and research settings. GAS involves parent–therapist goal setting of five child-specific goals which are scaled to five levels based on the child’s individual expected level of progress over a specified period (in this study, 3 months). GAS is an accepted and reliable measure for use with children with developmental challenges as it can capture individualized gains that are often not reflected or measurable on standardized outcome assessments. GAS provides subjective information about the client’s progress and measures the extent to which the client’s individual goals, set at the start of the intervention, are achieved because of the intervention. Goals are scaled using a 5-point scale (–2 to +2). Zero (0) identifies the predicted level of performance, –1 indicates somewhat less than expected outcome, −2 much less than expected outcome, +1 somewhat more than expected outcome, and +2 much more than expected outcome [48,49,50,51]. The goal scaling method used in this study was described by Kiresuk et al. (2014) [50], although different definitions for rating levels are used by other researchers.

### 2.4. Intervention

The HEP Approach intervention included weekly clinic-based parental coaching by a physical therapist for 3 months for a total of 12 one-hour sessions. All sessions were videorecorded. In addition, the family shared videos with their therapist once a week via the Whatsapp application and were supported via therapist feedback to complete home implementation of activities and suggestions presented in the clinic sessions. The treating therapist was trained and supervised by the first author, who developed the HEP Approach. The clinic-based intervention videos were reviewed weekly and feedback was given to the treating therapist by the first author to ensure sessions adhered to the key principles of the HEP Approach and maintained high fidelity standards.

During the HEP Approach intervention, the therapist and family cooperatively provided sustainable, continuous, individualized environmental enrichment to advance the active exploration and engagement of the child. Increasing parenting self-efficacy in the provision of enriched environmental conditions for the child is one of the most important building blocks in the HEP Approach. Therefore, the parent’s mindfulness, collaborative exploration, capacity building, self-regulation, empathic inquiry, and reflection were supported [52]. The HEP Approach consists of 10 essential intervention principles that are rooted in EE studies and defined by Balikci (2022): physiological homeostasis, safety, sensory experiences, spatial features of the environment, environmental and object novelty, challenge, enjoyment, continuous engagement, social opportunities, active engagement in and exploration of the environment. These key elements of the HEP Approach were implemented using core principles of Dynamical Systems Theory, Gibson’s Ecological Theory of Perception, Theory of Neuronal Group Selection, and Person–Environment–Occupation Model [4,20]. Table 1 presents definitions of the 10 essential features of the HEP Approach intervention and provides examples of how these principles were implemented in this case.

**Figure 1:**
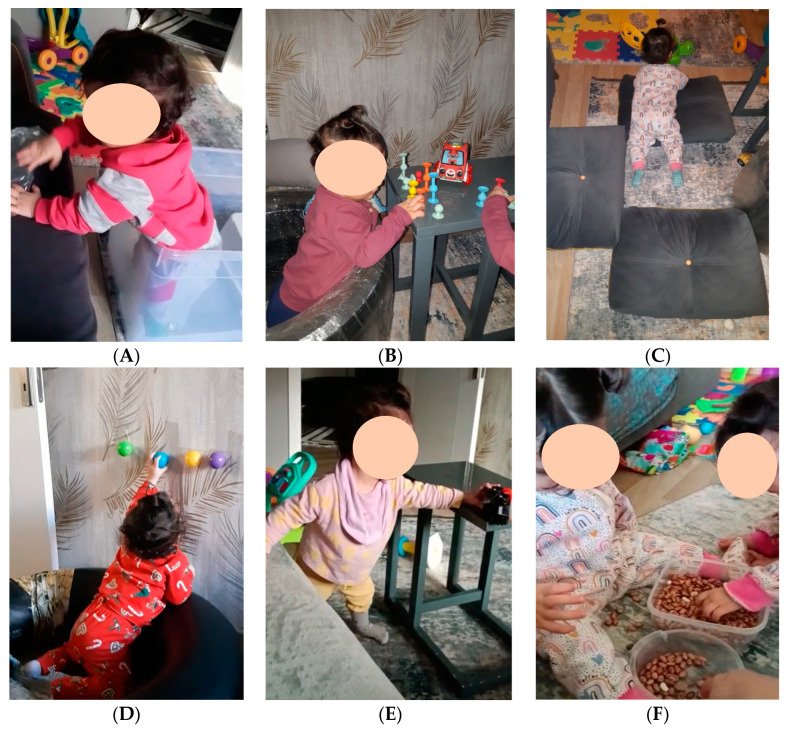
HEP Approach intervention. (**A**) Z is enclosed within a transparent plastic container, engaging with particular desired objects and exploring a range of possible actions with her body. The box provides a secure setting for her to explore objects and try different means of movement possibilities. Additionally, this support provided opportunities for Z to explore transitional movements from a seated posture to an upright posture. Proximity to the infant’s favored objects or toys acted as a stimulus to move the baby. (**B**) Arrangement of the space within the car tires to “just right” dimensions enabled Z to explore movement possibilities and objects while in a standing position without too much or too little support. In addition, her sister often accompanied her in this space, which provided support for Z’s drive and enthusiastic investigation of the objects within her reach. (**C**) Once Z acquired the skill of crawling, several objects were positioned on the room’s floor, prompting her to explore various ways of maneuvering around, over, or under them. The pieces underwent regular repositioning which provided a constant need for adaptive motor behaviors. (**D**) Enticing balls were placed on the wall to motivate Z to stand, and an inner tube was used as support to facilitate her exploration of the transition from seated to standing. (**E**) By positioning a compact table close to the sofa, Z was able to conveniently explore environmental opportunities for transitional postural movements while standing. Play activities were strategically arranged with her preferred toys to provide an incentive for her to transition between the table and the couch. (**F**) Z explored fine motor opportunities for manipulation while engaging in bean play. Her sister’s participation in these activities reinforced her motivation to engage in play and exploration within a social setting.

The therapist guided the mother to understand that homeostasis is the first step for enrichment and development. Exploring ways to recognize and reach homeostasis with her child was a key element of the clinical sessions. The significance of providing support to the infant by emphasizing the role of robust sensory systems to enhance perceptual abilities, and creating secure spatial and social environments that incorporate challenges and new experiences within the zone of proximal development for active exploration were highlighted. See Figure 1 for examples of home activities.

### 2.5. Analysis

Data collected included 11 data points using the PDMS-2: four for Phase A taken at 1-month intervals, six for Phase B taken at 2-week intervals, and 1 Follow-Up taken four months after the end of Phase B. Eight data points were collected for the TSFI: four for Phase A and three for Phase B, all taken at 1-month intervals and one for Follow-Up at four months after the end of Phase B. GAS goals were established at the start of Phase B and evaluated at the end of Phase B.

First, visual inspection and graphic representation of the data determined if baseline Phase A was stable for the PDMS-2 and the TSFI. The two-standard deviation Band (2SDB) method was used to determine if there were changes in the outcome measures during the intervention Phase B compared to the baseline Phase A and Follow-Up period. The mean of measures taken during the baseline Phase A +/− 2 standard deviation (2SD) was calculated. Intervention Phase B and Follow-Up phase data points that were greater than 2 SD above the mean were interpreted as a significant increase from baseline [41,53].

In addition, a second analysis used confidence intervals obtained from population data on the PDMS-2 (data was not available for the TSFI) as an acceptable means of determining if variability in pre–post scores was likely related to chance or not. A 95% confidence interval was applied to each score on the PDMS-2. Pre–post scores with non-overlapping confidence intervals reflect effect sizes greater than 1 and are interpreted as the results being unlikely to have occurred by chance, thus indicating a true change. Appropriate assessment manuals provided necessary confidence interval information [51,54]. 

To analyze the achievement of parent-identified GAS goals, the GAS scores were converted to a t-score. A t-score of 45 or higher indicates that the child has accomplished or exceeded the expected goal performance [49,50,51].

## 3. Results

**Figure 2:**
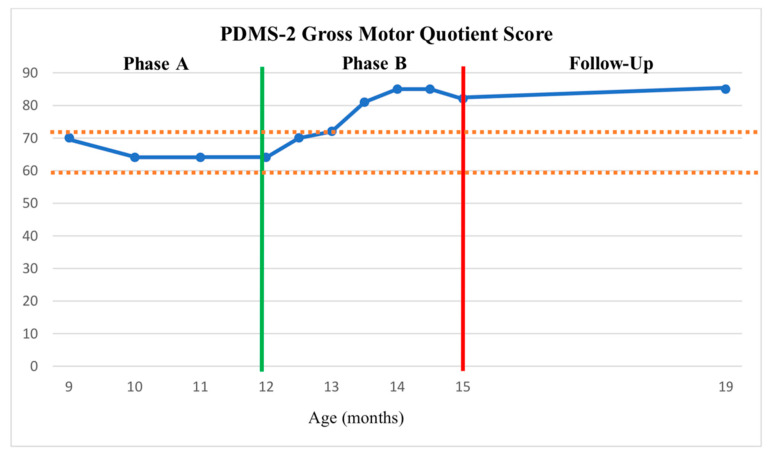
PDMS-2 Gross Motor Quotient (GMQ) scores across phases and Follow-Up as plotted about the +/− 2 SDB (between dashed lines). The green vertical line represents the start of the HEP intervention. The red vertical line represents the end of the HEP intervention.

**Figure 3:**
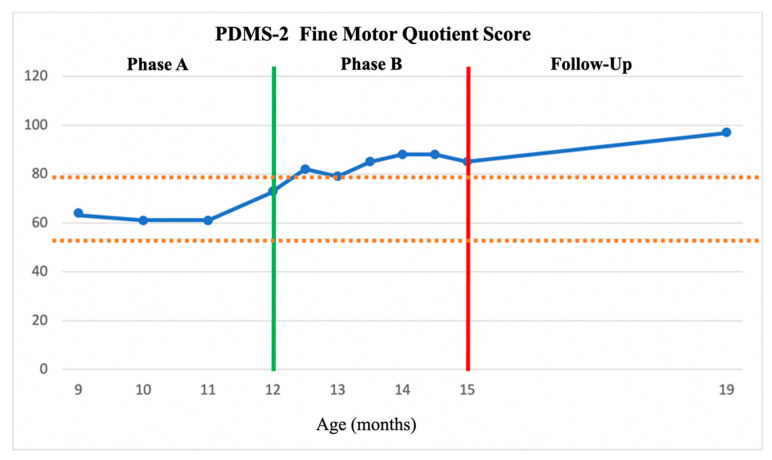
PDMS-2 Fine Motor Quotient (FMQ) scores across the AB phases and Follow-Up assessment. FMQ scores of PDMS-2 across the AB phases as plotted about the +/− 2 SDB (between dashed lines). A = The green vertical line represented the start of the HEP intervention. The red vertical line represented the end of the HEP intervention.

**Figure 4:**
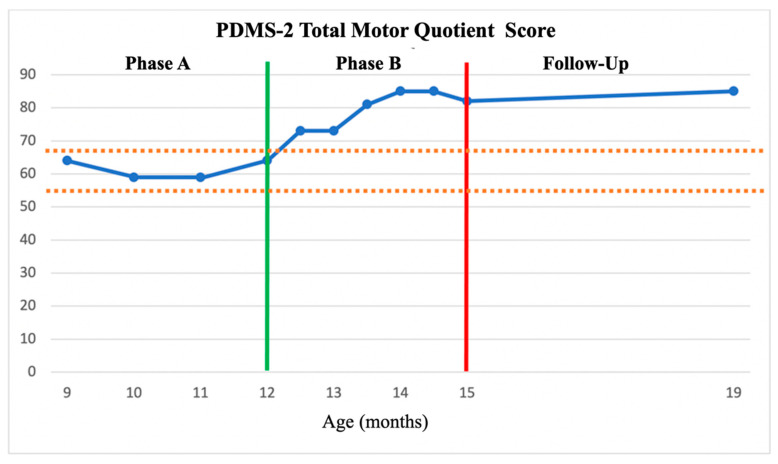
PDMS-2 Total Motor Quotient (TMQ) scores across the AB phases and Follow-Up assessment. TMQ scores of PDMS-2 across the AB phases as plotted about the +/− 2 SDB (between dashed lines). A = The green vertical line represented the start of the HEP intervention. The red vertical line represented the end of the HEP intervention.

Phase A—Baseline. Z completed all scheduled assessment and intervention sessions. No adverse events were reported. Stable baselines were documented on both the PDMS-2 and TSFI. The averages and standard deviations of Phase A for the PDMS-2 scores were M = 65.5 (SD = 3) for GMQ, M = 67.75 (SD = 5.67) for FMQ, and M = 61.5 (SD = 2.88) for TMQ. Applying the 2 SD Band Method, any score over M = 71.5 for GMQ, M = 79.09 for FMQ, and M = 67.26 for TMQ indicated change. No significant change in either measure was observed in Phase A which constituted the standard physical therapy intervention baseline for this study (see Figure 2, Figure 3 and Figure 4).

Phase B—HEP Approach Intervention. The PDMS-2 Phase B HEP Approach intervention scores averaged M = 79.33 (SD = 6.65) for GMQ, M = 84.50 (SD = 3.5) for FMQ, and = 79.8 (SD = 5.52) for TMQ, which are greater than the upper limit of the 2SDB for each score as established in Phase A. Five out of the six Phase B points for GMQ and FMQ, and six of the Phase B points for TMQ on the PDMS-2 were above 2SDB (see Figure 2, Figure 3 and Figure 4), thus indicating a significant improvement in Z’s gross, fine, and total motor outcome measures with the HEP intervention as measured by the PDMS-2 (see Figure 2, Figure 3 and Figure 4). Further, the GMQ and TMQ scores had no overlapping confidence intervals suggesting a significant improvement after HEP intervention in gross and total motor performance. The FMQ score confidence intervals overlapped by only one point (e.g., both included a score of 79); as this is a negligible overlap, it may represent a true change in fine motor performance (see Table 2). 

**Figure 5:**
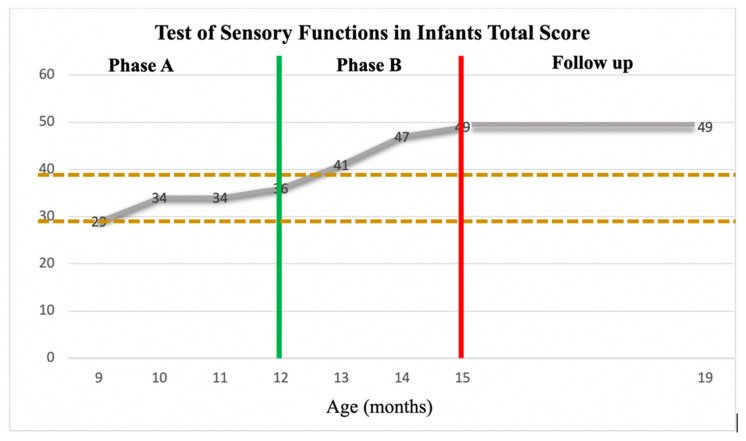
TSFI total scores across Phases A, B, and Follow-Up. TSFI total scores are plotted about the +/− 2 SDB (between dashed lines). The green vertical line represented the start of the HEP intervention. The red vertical line represented the end of the HEP intervention.

On the TSFI, the mean of the four TSFI total scores at Phase A was 33.25 (SD = 2.98). Thus, any score over 39.21 was above the 2SDB, representing a measurable change in the TSFI total score (see Figure 5). The TSFI Phase B scores averaged 45.66 (SD = 4.16) which was greater than the upper limit of the 2SDB (39.21) as established in Phase A. Thus, a significant change in Z’s overall sensory functions was found with the HEP Approach intervention (see Figure 5).

Z’s GAS goals, which were generated before intervention at the start of Phase B, were rated by her parents post-intervention at the end of Phase B. Z attained much more than the expected level of outcomes for goals 1–4 and demonstrated more than the expected outcome for goal 5 as shown in Table 3. This resulted in an overall GAS score of +1.8 which indicates that Z exceeded intervention expectations and demonstrated somewhat more progress than expected following the intervention. The GAS score of +1.8 translated to a t-score of 77.14 which reflected significant improvement on the goals. 

Follow-Up. Scores for both the PDMS-2 and the TSFI remained stable or improved following the HEP Approach intervention. The sustained improvement following the HEP intervention was also supported by the finding that the Follow-Up GMQ score, FMQ score, and TMQ score confidence intervals did not overlap with Pre-Phase A scores (see Table 2). Although Follow-Up assessment scores indicated that the rate of progress in gross motor performance and sensory processing skills slowed down, it can be suggested that general motor development and sensory processing were maintained. Fine motor skills continued to demonstrate improvement.

## 4. Discussion

These results found that the HEP Approach intervention resulted in significant progress over a traditional physical therapy program in improving Z’s motor skills and sensory functioning. Furthermore, Z demonstrated a somewhat more than expected outcome after the HEP Approach intervention in overall parental goals as measured by GAS.

Both the 2SD Band method and comparison analysis of overlapping confidence intervals demonstrated favorable motor development outcomes following the HEP Approach intervention. Nevertheless, there are discrepancies in certain areas between the outcomes of these two methodologies. Based on the 2SD Band method analysis, Z demonstrated a statistically significant improvement in all GMQ, FMQ, and TMQ domains following Phase B compared to Phase A. This improvement was maintained until the Follow-Up evaluation. However, the overlap of confidence interval analyses revealed a statistically significant improvement in GMQ and TMQ scores but not the FMQ scores. In all cases, this improvement remained stable throughout the Follow-Up period. During the Follow-Up period, the parents continued to provide home-based enrichment activities. However, because that 4-month time period was their summer vacation time, their usual routines were disrupted with travel, etc., resulting in less intense home intervention. It is possible that this accounts for fewer gains during Follow-Up than might have otherwise occurred. Future studies should examine the efficacy of follow-up improvements because of the parent training component of the HEP Approach intervention. Further, while existing EE approaches also incorporate a parental training component, the literature suggests that these approaches have typically been designed for specific developmental challenges, diagnoses, and/or age groups [55,56,57]. Therefore, clinicians often utilize the approach that is commonly used for the specific group they are treating. The HEP Approach is more comprehensive and is designed to apply to a wide range of populations and ages. It also is the only approach to incorporate all ten key components in a well-articulated manner.

The confidence interval overlap analysis suggested that the development of fine motor skills did not seem to make a statistically sufficient improvement. Similarly, Morgan et al. (2015) demonstrated in their study that GAME intervention improved only PDMS-2 TMQ scores, or overall motor skills, in infants with CP [58]. On the other hand, our fine motor quotient confidence intervals overlapped by only one point; again, this is a negligible overlap and may represent a functional change in fine motor skills. However, during the Follow-Up period, the FMQ score consistently increased, and the confidence intervals between the Follow-Up evaluation and the Post-Phase A evaluation did not overlap. This could be attributed to Z’s increased preference for manual exploration as her gross motor (especially sitting) skills developed [59,60,61,62]. 

Our findings align with studies that have demonstrated the beneficial impacts of EE-based interventions on the motor development of infants with CP [11,19,25,58]. In their study, Morgan et al. (2016) found that “GAME” (Goals—Activity—Motor Enrichment), an EE-based intervention model, is more effective than standard care in improving the motor function of infants who are at a high risk of developing CP [55]. Also, our previous case report demonstrated that the HEP Approach intervention promotes the motor development of an infant with CP [4]. A recent study revealed that the “Specific Task–Environment–Participation” (STEP) protocol, which is grounded on EE, yielded improved outcomes in improving particular motor skills in infants who are at risk of developmental delays when compared to the standard physiotherapy intervention [26]. Additionally, Apaydin et al. (2023) reported that the application of an EI program titled SAFE (Sensory Strategies, Activity-Based Motor Training, Family Collaboration, and Environmental Enrichment), which is based on EE, produced positive results for the motor development of premature infants [56]. Another study found that the 4-week EI program called CareToy training, which is influenced by the notion of an enriched environment and focuses on goal-oriented activities to encourage infants to complete specified tasks, can greatly enhance the motor and visual development of premature infants [57].

The HEP intervention improved Z’s sensory functions, and this progress persisted over the Follow-Up period. The manual of the TSFI did not provide confidence intervals, so we analyzed the change in sensory functions solely using the 2SD Band method. While motor skills have been examined in the existing body of literature, few studies examine the effect of EE-based interventions on the sensory functions of infants diagnosed with CP. However, in a previous study, we documented that the HEP Approach had a beneficial impact on sensory processing in a child with hemiparetic cerebral palsy [4]. Moreover, a recent study conducted by Apaydin et al. (2023) found that an EE-based intervention had beneficial effects on the sensory processing of preterm infants who are often at a high risk for CP [56]. The results of these studies align well with the present findings. A study conducted by Zhang et al. (2022) demonstrated that a targeted functional training program focused on improving motor development had a beneficial effect on the sensory processing abilities of preschool children, thus highlighting the importance of motor skills for the development of sensory processing skills [63]. Furthermore, the improvement in sensory functions in our study may be attributed to the infant’s greater engagement in active exploration and accompanying sensory experiences due to the advancement of motor skills [64,65,66]. 

Upon evaluating the 3-month intervention GAS goals established by the family, it was found that the child exceeded the expected level of achievement following the HEP intervention. Four of the goals achieved much more than expected outcomes, while only one goal achieved more than expected outcomes. Given that children with hemiparetic cerebral palsy typically tend to keep their afflicted hand closed [67], the improvement in this specific goal, which measures the frequency of maintaining the infant’s affected hand in an open position, is satisfactory. The outcomes of our study align with previous studies that demonstrate the impact of EE-based early intervention programs on the achievement of parental goals [55,58]. In their study, Morgan et al. (2015) demonstrated that the EE-based GAME intervention not only enhanced TMQ scores on the PDMS-2 but also resulted in positive improvements in the parental goals on the GAS [58]. In another study, the COPM was used to identify the primary goals parents have about their baby’s development. In addition, the COPM evaluated parents’ perspectives of their infants’ achievements for these goals, as well as their satisfaction with their infants’ present skills. The study revealed that parents of infants who underwent the GAME intervention for 16 weeks expressed a higher degree of satisfaction with the achievements accomplished by their infant [55]. 

These studies suggest that the HEP Approach may promote change due to a number of different mechanisms. First, empowerment of the family to provide continuous opportunities for enrichment for their child; second, the well-established neurological changes related to neuroplasticity as a result of enriched sensory–motor environments; and third, the child’s increased self-efficacy in interacting effectively and adaptively with the environment. The nature of these mechanisms and the explication of their process should be examined in further research studies.

The HEP Approach comprehensively addresses many areas of development including cognition, self-initiation, and social functioning that may be examined; however, this study only addressed two specific areas of function: motor development and sensory processing. This study did not assess these additional areas of development which presents a limitation to the efficacy findings of the approach. The lack of culturally adapted measures, especially for this age group, limited examination of these areas. Additional research addressing these outcomes would be useful. Further, as previously noted, future research should examine the parental training and carry-over aspect of this approach. A primary limitation of this study, though, is the methodological critique that is commonly directed at case studies in general (i.e., the limited applicability of a single case due to inadequate generalizability). Nevertheless, the objective of this case study is not to produce knowledge that can be applied broadly but rather to investigate the application of the HEP Approach, which incorporates the fundamental concepts of enriched environment paradigms utilized in experimental animal research, within the framework of ecological theories of human development with a child with a CP and TAPS diagnosis. Therapists may wish to consider the HEP Approach intervention as a way of supporting infants with TAPS who are at risk for CP and other developmental and neurological disorders.

## 5. Conclusions

Results of this study indicate that the HEP Approach intervention has the potential to improve sensory functions, motor skill outcomes, and parental goals in children with hemiparetic cerebral palsy who are under the age of two. Our work prompted a more comprehensive examination of the effectiveness of this intervention, which implements the concepts of EE to infants with cerebral palsy or those at risk, in an individualized manner, guided by ecological theories of development. Additional research on other areas of development, the role of the parental training aspect of the HEP Approach, and an explication of the proposed mechanisms of change for it would be useful in the future.

## Figures and Tables

**Table 1:** Definitions of the 10 essential features of the HEP Approach intervention and case examples.

Key Features of the HEP Intervention	Z Case Examples of Implementation of HEP Intervention
1. Homeostasis	The importance of Z’s sleep, feeding, and wakefulness cycles and general health status for her development were explained to her parents. It was explained that Z’s regulatory skills may also affect other developmental skills, and strategies to address regulation were suggested. For example, devices that supported Z’s independent movement (such as a baby walker) were recommended, or how to use a calm voice and a soothing touch when the baby struggled was demonstrated. Parents were educated on how their own well-being would affect the baby’s regulatory skills. Recommendations that they meet with friends or take a walk daily to support their own regulatory capacity were made.
2. Safety	The family was educated about the importance of Z feeling safe so that she can explore the environment, gain new experiences, learn, and develop. Examples included positioning in a rubber tube to help Z feel safe while sitting or moving to different positions. Or, giving verbal/emotional feedback to help Z feel safe when she gave signals that she was afraid.
3. Sensory Experiences	Caregivers were informed about Z’s sensory system strengths and challenges and their developmental importance. Environmental arrangements, body positions, and appropriate tools were provided so that Z could utilize her strong sensory systems. To support Z’s active exploration, arrangements of the environment were suggested for vertical positions (sitting, standing) in which she could use vision, one of her strong sensory systems (such as sitting in a transparent box). Arrangements to keep the affected limb within her visual field to improve her perception of it and, consequently, her active exploration using that limb were developed. Hearing, also one of the strong sensory systems, was used during movement and active exploration to improve the perception of what Z was doing through verbal feedback.
4. Spatial	The family was educated about the importance of active movement and exploration in a wider space. Support for Z’s active movement and exploration of physical space in her zone of proximal development was provided. For example, the use of a laundry box that provided optimum support surfaces so Z could actively explore mobility skills required for sitting while freeing her hands for engagement in potential interactions with the environment. Tools and arrangements were provided that allowed the child’s movement and active exploration in a larger space. For example, the furniture in the house was arranged so Z could hold on and move by cruising. The importance of visiting neighbors or relatives regularly and spending time in parks, etc., to help Z explore a wider space was also explained.
5. Novelty	The family was educated about the importance of novelty in experiences in development. Novelty was provided by adding small changes to active experiences with which Z was familiar. i.e., changing the direction of Z’s movement, toy location, or equipment placement in relation to Z’s position. Other examples included changing the position of furniture that provided support surfaces for active exploration and movement. A variety of bottles for drinking and differently shaped apple slices or crackers (cube, triangle, small, big) for self-feeding were used to promote novelty.
6. Challenge	The importance of providing challenges that Z could accomplish for development was explained to the caregivers. Appropriate motor challenge opportunities were explored by the parents for each situation with the guidance of the therapist. Examples included when Z was able to sit easily in a small laundry basket, then the use of a wider basket or box was explored by the parents. Or, when Z was able to cruise using the sofa, then the parents explored using the wall for cruising. The importance of providing challenges in all other developmental areas was also emphasized. For example, to support the social–emotional domain, opportunities were created for Z to interact with unfamiliar adults using familiar gestures. Or, when the baby was able to place pegs in a peg board, cognitive challenges were presented with activities aimed at placing simple shapes in their slots. Problem-solving challenges such as removing colored beans inside a water bottle were also suggested.
7. Enjoyment	The importance of fun and motivation in Z’s learning and development was emphasized. For this purpose, the family took care to choose toys or organize games that motivated Z. In addition, they supported Z’s motivation by including her twin in similar activities.
8. Continuous Engagement	The importance of opportunities for Z’s continuous active engagement, exploration, and movements in the environment and with others at all times in terms of facilitating development and the fundamental role of the family was emphasized. For this reason, instead of making suggestions to the family, as much as possible, opportunities that supported Z’s development, were suitable for her profile, and were compatible with the family dynamics were elicited from the family itself. Reflective questions such as “What do you think could be the reason why Z prefers this toy?” supported the family’s reasoning and problem-solving skills. Thus, the family supported the continuity of Z’s active exploration through the provision of appropriate opportunities at home.
9. Social	Optimum ways of promoting social relationships with Z were explored by watching the infant and asking reflective questions of the parents. The family learned how fewer words, shorter sentences, and more gestures and mimicry could be used effectively. For more social relationship opportunities, it was recommended that the family make more frequent visits to neighbors and relatives. In addition, the family identified that it would be effective for them to be a bridge to support Z’s interaction with others, i.e., the mother explained other’s behaviors to Z, and then her behaviors to others.
10. Active Engagement and Exploration	The importance of people around the child, the space she inhabits, and the equipment/materials available to encourage Z’s active exploration was emphasized. Allowing enough time for active exploration for each twin was explained. With support from the therapists, the parents explored the most appropriate spaces, equipment, tools, communication, and natural reinforcers to facilitate Z’s active exploration. For instance, they proposed that if the twin sister were to join Z, it would serve as an inherent motivator for engagement in active inquiry. Another suggestion made by parents was to use bigger toys to support bimanual exploration.

**Table 2:** The PDMS-2 gross motor, fine motor, and total scores with 95% confidence intervals.

PDMS-2 Scores	Quotient Score				95% Confidence Interval			
	Pre-Phase A	Post-Phase A	Post-Phase B	Follow-Up	Pre-Phase A	Post-Phase A	Post-Phase B	Follow-Up
GMQ	70	64	83	79	64–76	58–70	77–89 ^a^	73–85 ^b^
FMQ	64	73	85	97	58–70	67–79	79–91	91–103 ^b^
TMQ	64	64	82	85	60–68	58–70	76–88 ^a^	79–91 ^b^

Note. Pre-Phase A and Post-Phase A demonstrated no significant change. Significant changes among Post-Phase A and Post-Phase B scores are indicated with an “a”. Significant changes among Post-Phase A and Follow-Up scores are indicated with a “b”.

**Table 3:** Goal Attainment Scale post-intervention.

Goal	Much Less Than Expected Outcome	Somewhat Less Than Expected Outcome	Expected Outcome	Somewhat More Than Expected Outcome	Much More Than Expected Outcome
1.	Z will be able to manipulate toys less than 30 s while sitting supported by pillows.	Z will be able to manipulate toys for 30 s while sitting supported by pillows.	Z will be able to manipulate toys for 60 s while sitting supported by pillows.	Z will be able to manipulate toys for 90 s while sitting supported by pillows.	**Z will be able to manipulate toys for 120 s while sitting supported by pillows.**
2.	Z attempts to assume a 4-point crawling position and achieves this position on her own 1–2 out of 10 attempts.	Z attempts to assume a 4-point crawling position and achieves this position on her own in 3–4 out of 10 attempts.	Z attempts to assume a 4-point crawling position and achieves this position on her own 5–6 out of 10 attempts.	Z attempts to assume a 4-point crawling position and achieves this position on her own 7–8 out of 10 attempts.	**Z attempts to assume a 4-point crawling position and achieves this position on her own 9–10 out of 10 attempts.**
3.	Z is unable to stand supported by her parents and bear weight on her feet.	Z stands supported by her parents and bears weight on her feet for 1–5 s.	Z stands supported by her parents and bears weight on her feet for 6–10 s.	Z stands supported by her parents and bears weight on her feet for 11–15 s.	**Z stands supported by her parents and bears weight on her feet for 16–20 s.**
4.	Z is unable to manipulate objects with her affected hand even if the affected upper extremity is positioned in front of the body.	Z manipulates objects with her affected hand spontaneously 1–2 out of 10 attempts when the affected upper extremity is positioned in front of the body.	Z manipulates objects with her affected hand spontaneously 3–4 out of 10 attempts when the affected upper extremity is positioned in front of the body.	Z manipulates objects with her affected hand spontaneously 5–6 out of 10 attempts when the affected upper extremity is positioned in front of the body.	**Z manipulates object with her affected hand spontaneously 7–8 out of 10 attempts when the affected upper extremity is positioned in front of the body.**
5.	Z’s affected hand is never (0% of the time) open.	Z’s affected hand is rarely (25% of the time) open.	Z’s affected hand is occasionally (50% of the time) open.	**Z’s affected hand is frequently (75% of the time) open**.	Z’s affected hand is always (100% of the time) open.

Note. Bold areas indicate the level of achievement as rated post-HEP intervention.

## Data Availability

All the data are included in the manuscript.
